# Ion beam profiling from the interaction with a freestanding 2D layer

**DOI:** 10.3762/bjnano.8.73

**Published:** 2017-03-23

**Authors:** Ivan Shorubalko, Kyoungjun Choi, Michael Stiefel, Hyung Gyu Park

**Affiliations:** 1 Laboratory for Reliability Science and Technology, Empa (Swiss Federal Laboratories for Materials Science and Technology), Überlandstrasse 129, CH-8600 Dübendorf, Switzerland; 2 Nanoscience for Energy Technology and Sustainability, Department of Mechanical and Process Engineering, Eidgenössische Technische Hochschule (ETH) Zürich, Tannenstrasse 3, CH-8092 Zürich, Switzerland

**Keywords:** exposure dose, focused ion beam, freestanding 2D layer, graphene, ion beam diameter, ion beam point spread function

## Abstract

Recent years have seen a great potential of the focused ion beam (FIB) technology for the nanometer-scale patterning of a freestanding two-dimensional (2D) layer. Experimentally determined sputtering yields of the perforation process can be quantitatively explained using the binary collision theory. The main peculiarity of the interaction between the ion beams and the suspended 2D material lies in the absence of collision cascades, featured by no interaction volume. Thus, the patterning resolution is directly set by the beam diameters. Here, we demonstrate pattern resolution beyond the beam size and precise profiling of the focused ion beams. We find out that FIB exposure time of individual pixels can influence the resultant pore diameter. In return, the pore dimension as a function of the exposure dose brings out the ion beam profiles. Using this method of determining an ion-beam point spread function, we verify a Gaussian profile of focused gallium ion beams. Graphene sputtering yield is extracted from the normalization of the measured Gaussian profiles, given a total beam current. Interestingly, profiling of unbeknown helium ion beams in this way results in asymmetry of the profile. Even triangular beam shapes are observed at certain helium FIB conditions, possibly attributable to the trimer nature of the beam source. Our method of profiling ion beams with 2D-layer perforation provides more information on ion beam profiles than the conventional sharp-edge scan method does.

## Introduction

Focused ion beams (FIBs) have been increasingly exploited in nanotechnology for more than 40 years [1]. One of the most important parameters of FIBs in this respect is the beam diameter near the focal point. The most commonly used method for estimating the beam size is measuring a characteristic signal change (typically the amount of generated secondary electrons) when the beam is scanned over a very sharp sample edge. This method is called rise-distance or sharp-edge (knife-edge) scan method and has been adopted historically from a profiling technique of focused electron beams [2]. For the electron beams this method turned out to be very successful and could even be automatized to determine the diameter and astigmatism [3]. Nevertheless, this method turned out to be unwieldy in the ion beam profiling. Even in the initial attempts to adopt the sharp-edge scan method for precise measurements of the beam diameters of FIBs with energies of few tens of kiloelectronvolts, a number of experimental pitfalls have been detected [4–7]. First of all, actual sharp edges have variations in morphology, size and angle, thus resulting in diverse yields of secondary electrons. Second, scanning a focused ion beam across the knife edge can change the edge shape because of a milling effect incurred by the ion beam irradiation itself. Increasing the scan speed over the edge in order to avert the damage, gives rise to other problems such as shot noise and statistical beam fluctuations. Another strategy to measure FIB profiles is to exploit the milling effect of a bulk substrate by ion beams with a controlled beam dose. This method was actually used to estimate the focus spot size of one of the first Ga-FIBs [8]. In order to extract the ion beam profiles from such experiments more precisely, one needs to go through a complicated conversion from a beam shape to a milled pattern shape. In addition, scanning electron microscopy has to be accompanied with atomic force microscopy for more precise profile measurements of the milled nanostructures [9–12]. Commercially available FIB systems are usually provided with spot-size specifications defined via imaging resolution. One should pay attention to the fact that the resolution in FIB imaging depends mainly on the narrowest part of the beam, which can comprise only the top 10% of the current density distribution. Hence, it is practically impossible to extract information on the beam tails in this way. With the rise of graphene [13] and technological developments of creating suspended graphene it becomes possible to study its interaction with FIBs [14–16]. The key property of freestanding graphene in this case is its atomic thickness. FIBs interaction with such a self-suspending layer brings no collision cascade. Instead, atoms of the 2D membrane material are sputtered only by direct binary collision events with incident ions. Thus, the patterning resolution is directly set by the beam diameters. In fact, it was shown that FIB interaction with 2D materials contains information about beam profiles [14,17–18]. Here we show that it is possible to fabricate pores in graphene membranes smaller than the ion beam diameter by carefully tailoring the exposure dose. The pore diameters directly depend on the time for which individual pixels are exposed to ion beams, and in return this dependency reflects information of the ion beam profile. We determine a Ga-FIB point spread function and verify its Gaussian profile for different beam current values. The volume under the Gaussian profile is used to extract the graphene sputtering yield in good agreement with previously reported values [14]. Helium focused ion beams are characterized in a similar way to determine the previously unknown profile and possible asymmetries in it. Under certain conditions triangular He beam shapes are observed, possibly attributable to the trimer nature of the beam source. For imaging the milled pores we use scanning electron microscopy (SEM), scanning transmission electron microscopy (STEM) and helium ion microscopy (HIM). All methods give similar results regarding the measured focused ion beam profiles. Finally, we discuss technical limitations and critical steps towards ion beam profiling using this method.

## Results and Discussion

One of the most crucial aspects of ion beam profiling via the direct interaction with suspended graphene is the preparation of the ultraclean graphene membranes. First, graphene was grown on a copper foil using chemical vapor deposition (CVD) according to a previously developed method [19]. This method is optimized for maximal grain connectivity resulting in uniform graphene films. Then, graphene was transferred to silicon/silicon-nitride frames with openings of a few micrometers in diameter. A PMMA-based graphene transfer method with copper foil etching in ammonium persulfate was used. After the transfer graphene membranes are cleaned by annealing at 400 °C in hydrogen/argon atmosphere (900 sccm/100 sccm) for 60 min. As a result clean freestanding graphene membranes are obtained [14–15].

**Figure 1:**
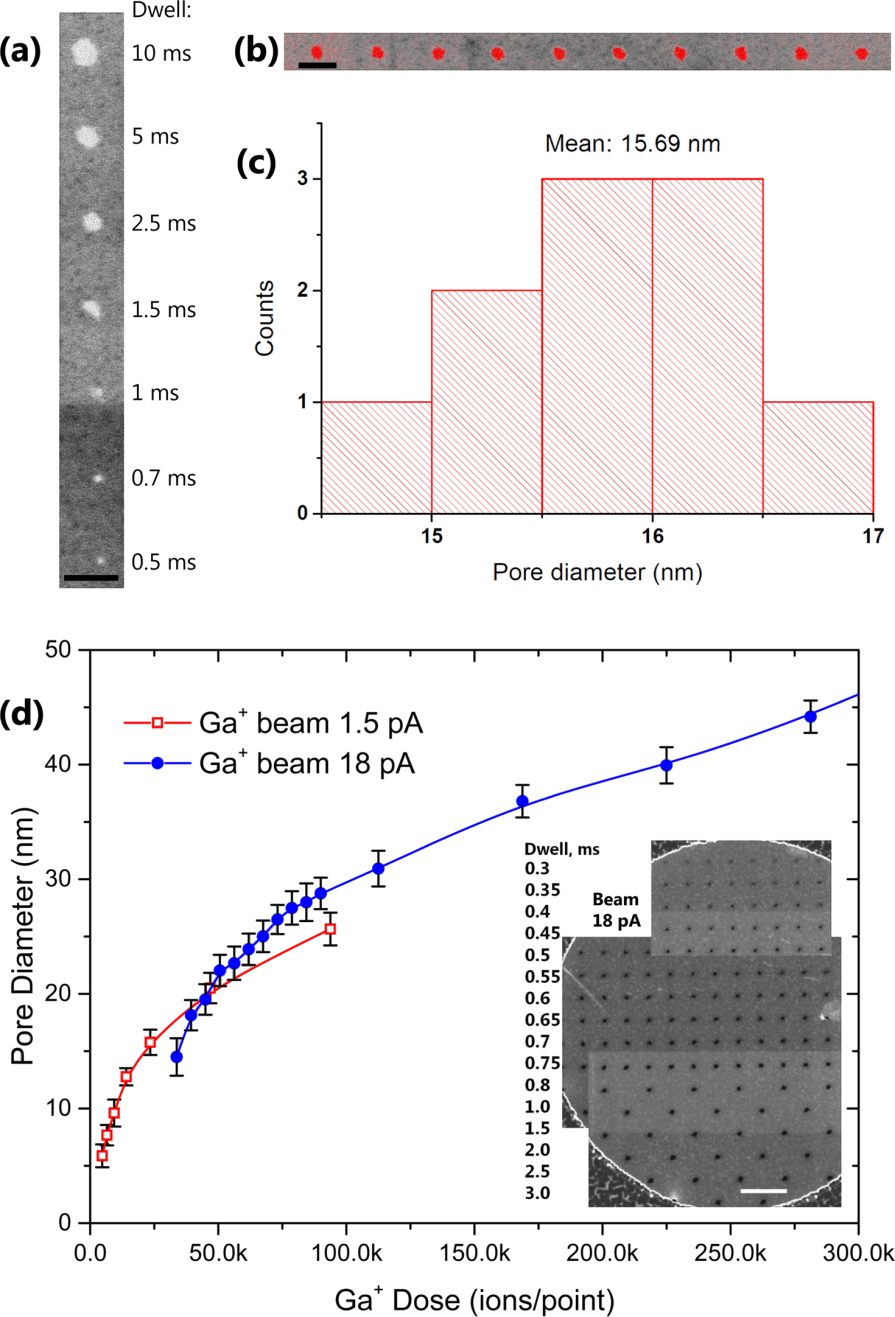
(a) STEM-BF image of a graphene membrane perforated with a 1.5 pA Ga-FIB. Pores diameter increases for longer dwell time. Scale bar is 50 nm. (b) Ten pores created under the same conditions, beam current 1.5 pA and dwell time 2.5 ms per spot. The red color indicates the pore areas used to calculate the diameters. Scale bar is 50 nm. (c) A histogram of pore diameters extracted from (b). (d) Graphene pore diameter dependency on Ga-FIB dose for different beam currents. Dots represent extracted mean values from the corresponding dose histograms, as shown in (c). Lines are drawn for visual guidance. Inset shows STEM-DF image of perforated graphene with 18 pA Ga-FIB. Pores within the same row are created with the same dwell time, indicated to the left of the image. Scale bar is 200 nm.

As a first step, freestanding graphene membranes were exposed to a Ga^+^ focused ion beam. The smallest beam aperture gives a beam current value of 1.5 pA. A suspended graphene layer is then exposed in a single-pixel exposure mode. Figure 1a shows a STEM bright field (BF) image of seven pores milled into graphene with different dwell times ranging from 0.5 to 10 ms. A clear dependence of the pore size on the exposure dose is observed. It is also seen that pores can have slightly irregular shapes. In order to precisely extract pore diameters from such experiments, we perforated sets of pores with identical exposure parameters. Figure 1b demonstrates an example of an array of 10 pores milled with a 1.5 pA beam for 2.5 ms each. ImageJ [20] is exploited for automatic contrast detection and extraction of the pore area indicated in red. The diameters are calculated from the pore areas, assuming a round shape of the pores. Figure 1c shows the corresponding histogram of the pore-diameter distribution for the ten pores. Mean values and standard deviations are extracted from these histograms. This method includes control over statistical variations and allows for precise pore-diameter measurements. Throughout this paper we use this method to extract pore-diameter values for each parameter set of the ion beam exposure. Figure 1d shows the dependency of the pore diameters on the Ga^+^ ion dose extracted from beam current and dwell time.

Data for two instrument apertures corresponding to two different beam currents are presented: squares for 1.5 pA and circles for 18 pA beam current. STEM dark field (DF) images of a pore array created with 18 pA beam current are shown in the inset of Figure 1d. The pore diameter vs ion exposure dose curves have very distinct shape Figure 1d. Pore diameters steeply increase for the low exposure doses and then gradually increase for larger doses. For 1.5 pA Ga-FIB current and 0.5 ms dwell time pores with diameters as small as 5 nm could be reliably created and identified. Dwell times of 0.3 ms resulted in a large statistical variation of pore diameters: from a few nanometers down to no pores observed in some cases. For such small pores, STEM resolution comes to its limits. An intuitive explanation of the behavior observed in Figure 1d can be visualized as follows: In contrast to FIB interaction with bulk objects no collision cascade exists in the case of suspended 2D layers. Carbon atoms of the graphene membrane will be sputtered only to the direction of the incident ion momentum. When Ga^+^ ions with 30 keV energy hit the graphene membrane, carbon atoms are sputtered at a probability of about 50% [14]. Ga-FIBs are known to have Gaussian beam profiles to a large extent [4–6]. First, the rather flat top part of the beam profile will create enough collision events with graphene to open the initial pore. This process is unstable and very sensitive to the dwell time. Thus, initial pore formation can be seen as a stochastic process. Once the initial pore is created the graphene material around it will be further removed by the bombarding ions within the Gaussian bell. The growth of the pore diameter with dwell time is eventually slowed down, thereby reflecting the tail of the beam profile. The pore growth dynamics is defined very precisely by the incident ion beam distribution. Thus, the point spread function (PSF) of the incoming FIB can be extracted from the dependence of the pore diameter on the dwell time. In a similar way it was suggested to extract the PSF of He FIB from exposed resist shapes [21]. The main difference is that in case of interaction between ion beam and resist there is a finite volume involved, resulting in a less precise transformation from the shape of the exposed resist to the PSF of the beam. The smallest PSF found in this way was at least 10 nm wide, several times larger than the specified He^+^ beam diameter [21]. In the case of the suspended 2D layer the finite interaction volume is minimized to only one atomic layer, which ultimately sets the closest relation between the patterns created and the beam shapes.

**Figure 2:**
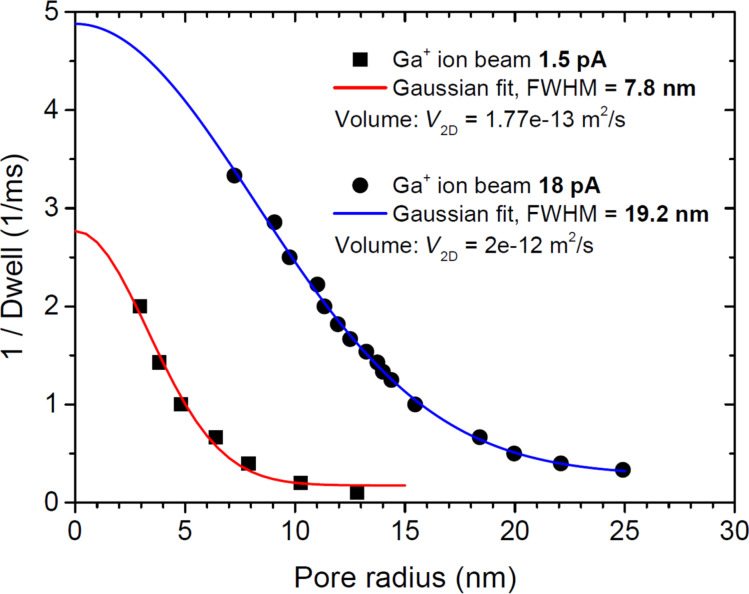
Gaussian fits to the 1/dwell vs pore radius curves. FWHM and volume under the Gaussian lines are extracted for both Ga ions beam currents: 1.5 and 18 pA.

When focused ion beams with a certain PSF irradiate suspended graphene for a time period (dwell time), a part of the graphene layer where the exposure dose is higher than a critical dose value (defined by sputtering yield) will be removed to yield a pore of a certain radius. Therefore, a plot of inverted dwell time vs pore radius would represent the PSF of the beam. Figure 2 shows such a plot for different Ga^+^ beam currents used in Figure 1d. For fitting Gaussian functions to the experimental data the following relations are used:


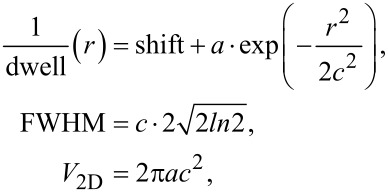


where, *r* is the pore radius, *a* is the height of the peak, *c* is the inflection point of the Gaussian curve, FWHM is the full width at half maximum, and *V*_2D_ is the volume under the Gaussian bell. The “shift” parameter represents an offset of the Gaussian peak from zero and originates mainly from non-Gaussian tails of the beam. As can be seen from Figure 2 the Gaussian fitting function works very well; the coefficients of determination (*R*^2^) are higher than 0.98 for both curves. The FWHM for the 1.5 pA Ga-beam is extracted to be 7.8 nm. Specifications of the instruments give 7 nm at 1.1 pA. Actually, to get this low beam current the smallest aperture has to be used. In our case the aperture was slightly worn out resulting in a higher current. This would explain the small deviation of measured FWHM from the specifications of the instrument. Interestingly, the imaging resolution with such a beam is specified to be 4 nm (determined from 35/65 edge contrast), which means that the current of less than the top 20% of the beam profile defines the imaging resolution. Another important parameter of the Gaussian curve is its bell volume, *V*_2D_. For the 1.5 pA beam current, *V*_2D_ = 1.77 × 10^−13^ m^2^/s. This figure can be seen as the amount of etched graphene area per second. Knowing the atomic density of carbon in graphene, σ_C_ = 3.82 × 10^19^ m^−2^, one can calculate the amount of removed carbon atoms per unit time *N*_C_/*t* = *V*_2D_·σ_C_ ≈ 6.76 × 10^6^ s^−1^. At the same time the number of incident Ga ions per unit time can be calculated as beam current divided by elementary charge, *N*_Ga_/*t* = *I*_beam_/*e* = 9.4 × 10^6^ s^−1^. By relating the number of sputtered carbon atoms to that of incident Ga^+^ ions, we can estimate sputtering yield of graphene, γ_C/Ga+_ = *N*_C_/*N*_Ga+_ ≈ 0.7. In reality, the removal of carbon atoms from the lattice leads to a lower probability of collision events during subsequent ion incidence. Thus, it leads to the decrease of sputter yield with increasing ion irradiation dose, as described in [14]. The obtained value of the sputtering yield here agrees very well with the one determined experimentally and calculated from the binary collision model in [14]. A similar analysis can be made for 18 pA Ga^+^ beam current. In this case we measure the FWHM of the beam PSF to be 19.2 nm, which is larger than the specification of 13 nm. The graphene sputtering yield estimated in the same way as above gives γ_C/Ga+_ ≈ 0.68. The fact that all numbers agree well with each other strengthens the model used for understanding the underlying physical phenomena. The method can be automated and used as a quick way to precisely measure Ga beam profiles in dual-beam systems.

**Figure 3:**
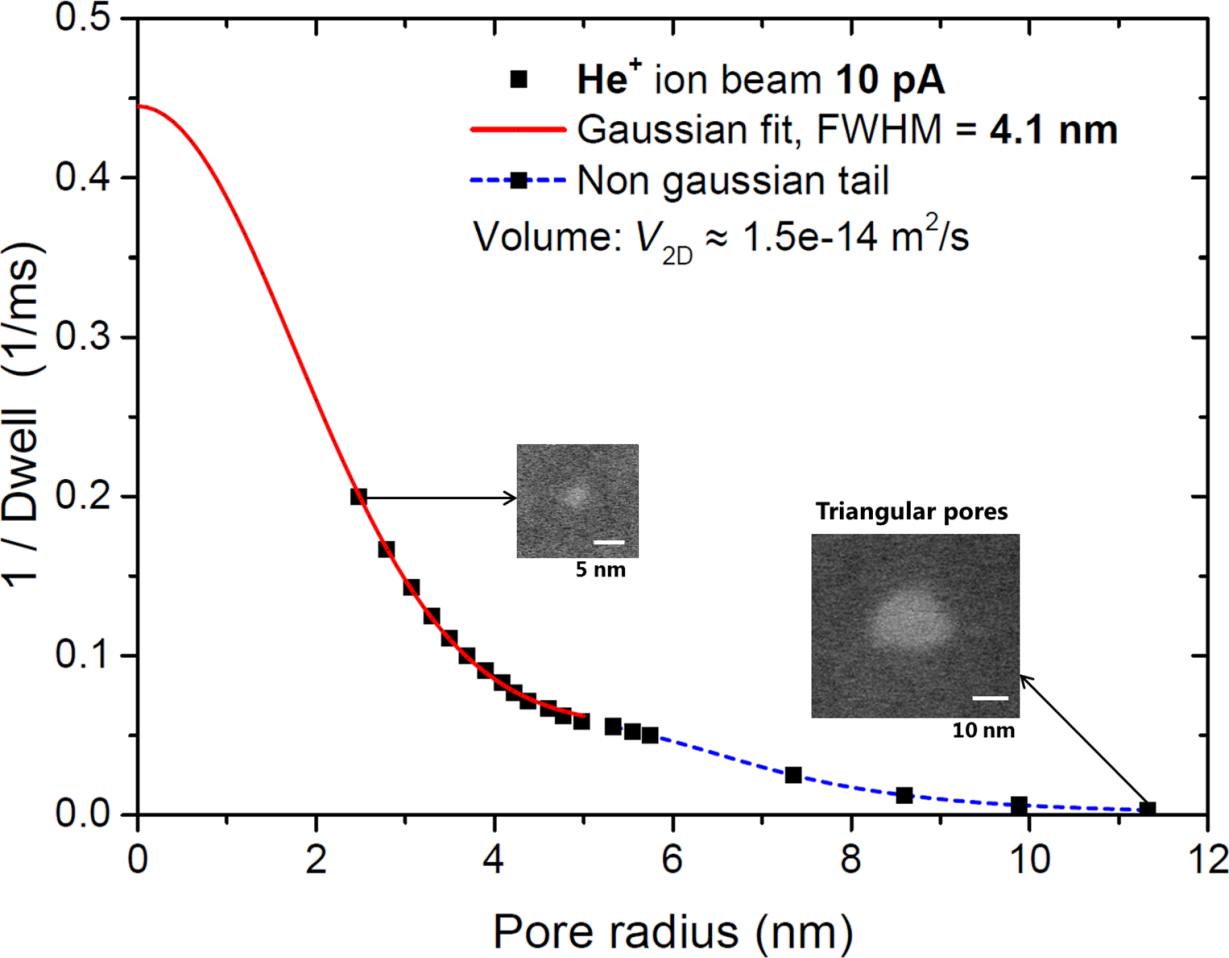
10 pA Helium ion beam profile extracted from graphene perforation experiments. The main peak can be fitted well with a Gaussian shape. Insets are STEM images of the pores made by main part of the ion beam and its tail. Note a triangular pore shape created by long exposures.

As the next step, we perform similar experiments with the helium focused ion beam. He FIB has several conceptual differences compared to the Ga FIB. It is based on a gas-field ion source (GFIS) that is disparate from a liquid metal ion source for gallium. GFIS features an ultra-sharp tip ending with a stable configuration of only three atoms, known as a trimer ionizing helium atoms in its vicinity [22]. This sharp source is a key feature for obtaining the high resolution in HIMs. The second important difference from Ga^+^ ions is the low mass of He^+^ ions which results in much smaller sputtering yields [14–15]. We choose 10 pA beam current for the experiment here. This high current is relevant for increasing the He-FIB nanopatterning speed. It is also expected to have a large enough beam diameter to be resolved in the presented imaging methods. Arrays of pores in freestanding graphene are created by exposing single pixels with different dwell times between 1 and 1000 ms. The 1/dwell vs pores radius data is plotted in Figure 3. As one can see, this particular case does not have a smooth peak curve. The two STEM images insets in the figure show the differences in shape of the pores. Pores created with long exposure times have a distinct triangular shape. This behavior is reproducible and is observed also for higher He-FIB currents. Nevertheless, for He-beam currents smaller than 1 pA only round pores were observed even for long exposure times. Technically, the current in the He-FIB is tuned by changing a parameter called “spot”, which determines the size of the beam at aperture position. For smaller currents only part of the beam extracted from the source passes through the aperture, ideally from one of the trimer atoms. For larger currents, a fraction of the beam passing through the aperture is larger. Thus, it includes also current generated by all three atoms of the trimer. In this way PSF of the beam can have triangular shape because of the trimer nature of the GFIS. In respect to the topic of this paper, we find this result very interesting because it clearly demonstrates the ability of our method to measure beam profiles of arbitrary shapes. In contrast, the standard sharp-edge scan method would not give information about triangular profile of the beam.

Strictly speaking, it is not the best way to present triangular pores in the plot where the characteristic length scale of the pores is radius. Nevertheless, we calculate the effective radius of the pore from the area of a triangular pore and assuming its round shape for completeness of the curve in Figure 3. Clearly, the tail where triangular pores are observed does not match the Gaussian fit. Thus, we limit Gaussian fit only to the main peak, shown by the red solid line in Figure 3. Actually, the data fit very well to the Gaussian distribution within the main part of the beam with the coefficients of determination being larger than 0.99. The FWHM of the Gaussian part of the beam is measured to be 4.1 nm. Usually, HIMs are operated at 0.5 pA beam current giving an imaging resolution below 0.5 nm. Our measurement suggests that at a beam current of 10 pA, the imaging resolution can still be as good as a few nanometers.

As the next step we analyze the volume under the PSF of the 10 pA He beam. The total volume including Gaussian fit and a tail is estimated to be *V*_2D_ = 1.5 × 10^−14^ m^2^/s, the resulting graphene sputtering yield is γ_C/He+_ = *N*_C_/*N*_He+_ ≈ 0.09. This value agrees well with the previous report [14].

As a final remark we discuss the limitations of the described method to measure the profiles of energetic focused ion beams. First, cleanliness of the graphene membranes is very important. Membranes thicker than monolayer graphene will not give an accurate relation between pore radius distribution and beam profile. Also if graphene is contaminated with organic molecules, these will decompose during the ion beam irradiation or exposure to secondary electrons and will deposit as amorphous carbon on the graphene surface competing with the sputtering process. This will result in an incorrect determination of dwell time and false beam profile curves. The second important issue is high-resolution imaging of the pores. When pore size becomes smaller than a few nanometers, like in the case of 1–3 pA He^+^ beams, it is difficult to measure these pore dimensions precisely with standard STEM or HIM. Time consuming high-resolution TEM imaging would be required. The third observed limitation is the graphene membrane collapse under high current Ga^+^-FIBs. Actually, in the inset of Figure 1d one can see that for longer exposure times the density of the pores is deliberately reduced. This is because of mechanical breakdown of the pores under large irradiation currents and doses. Thus, measuring Ga^+^ beam profiles of currents higher than a few hundred picoamperes would be difficult with the graphene-irradiation method because of the mechanical stability of the layer.

## Conclusion

Precise measurement of the profiles of energetic focused ion beams from their interaction with suspended graphene is demonstrated. Dependency of pore dimensions of the milled-in graphene on the exposure dose reveals information on a point spread function of the incoming ion beam. The method gives more information on the ion beam profiles than the conventional sharp-edge scan method.

## Experimental

Graphene was grown using CVD on an Alfa Aesar 46986 Cu foil. Before the growth the foil is cleaned by Ar ion beam milling for 10 min at 250 mA and 600 V. Then, it is reduction-annealed in a H_2_/Ar gas flow (5 sccm/5 sccm) at 1000 °C for 60 min. The growth of graphene is initiated by introducing a CH_4_ gas flow (10 sccm) for 40 min.

FEI Helios NanoLab G3 UC DualBeam SEM/Ga-FIB system and Zeiss Orion Plus He-FIB equipped with a Raith Elphy MultiBeam pattern generator were used for the presented study. Both, Ga and He-FIBs were operated at 30 kV acceleration voltage and ca. 6 × 10^−5^ Pa chamber pressure. The smallest aperture (8 μm) was used for the Ga-FIB to obtain the 1.5 pA beam current, and the third smallest aperture (25 μm) was used for the 18 pA current. He-ion beam current selected for this work was 10 pA. It was defined by the following hardware parameters: 10 micrometer aperture, 6.7 × 10^−4^ Pa helium pressure in the gun chamber, and a “spot” parameter between 1.8 and 2.5. SEM in the DualBeam device was used in STEM bright-field and dark-field mode (BF and DF) at 30 kV and 50 pA probe current for perforated graphene imaging. HIM at 30 kV, 0.5 pA beam current, 1 μs dwell time and 8–32 lines averaging was used for pores imaging. The pixel resolution was chosen to be better than 0.5 nm/pixel.
